# Supplementary material for: Content-rich biological network constructed by mining PubMed abstracts
Source: BMC Bioinformatics. 2004 Oct 8;5:147. doi: 10.1186/1471-2105-5-147 (PMC528731; doi:10.1186/1471-2105-5-147)
Supplement: Additional File 2 — The original results of the above study (non-essential files are deleted to keep the file size under the limit set by BMC bioinformatics). [file 1471-2105-5-147-S2.bz2 › chilibotAdditionalFile2/dip05/87ID7720710E323/html/POU2F2_HMGB2.html]

 


 **POU2F2** and **HMGB2** 
  
Found 1 abstracts in PubMed, retrieved 1.  
 

 What does Google say? 
 PDF only 
| .edu only 

---

**Interactive relationship** (e.g. stimulation, inhibition, etc)

**Neutral relationship**- We have mapped the interaction domains for both proteins and have shown that HMG2  [ **HMGB2** ]  and Oct2  [ **POU2F2** ]  interact via their HMG domains and POU homeodomains, respectively.  Ref: 7720710 EMBO J, 1995
- This interaction is not restricted to Oct2  [ **POU2F2** ] , as other members of the octamer transcription factor family like Oct1 and Oct6 also interact with HMG2  [ **HMGB2** ] .  Ref: 7720710 EMBO J, 1995
